# Abnormal Origin of the Left Coronary Artery: When the Bloodstream Finds its Way

**DOI:** 10.5334/jbsr.3085

**Published:** 2023-04-10

**Authors:** Amin Mahsouli, Cristina Anca Dragean

**Affiliations:** 1 Cliniques Universitaires Saint-Luc, UCL, BE

**Keywords:** ALCAPA syndrome, LCA steal phenomenon, coronary anomaly, myocardial infarction

## Abstract

**Teaching Point:** Anomalous origin of the left coronary artery from the pulmonary artery (ALCAPA syndrome) is a rare congenital anomaly characterized by a steal phenomenon; it must be recognized, as a prompt treatment may prevent life-threatening complications such as myocardial infarction, mitral dysfunction, and malignant dysrhythmias in adults.

## Case History

A 46-year-old female with recurrence of a persistent atrial fibrillation (PAF) was sent to our department to perform cardiac computed tomography (CT) before a cardiac ablation. The patient’s relevant history was mitral valvuloplasty and several cardiac infarcts. No cardiac insufficiency was found on the cardiac evaluation.

**Figure 1:**
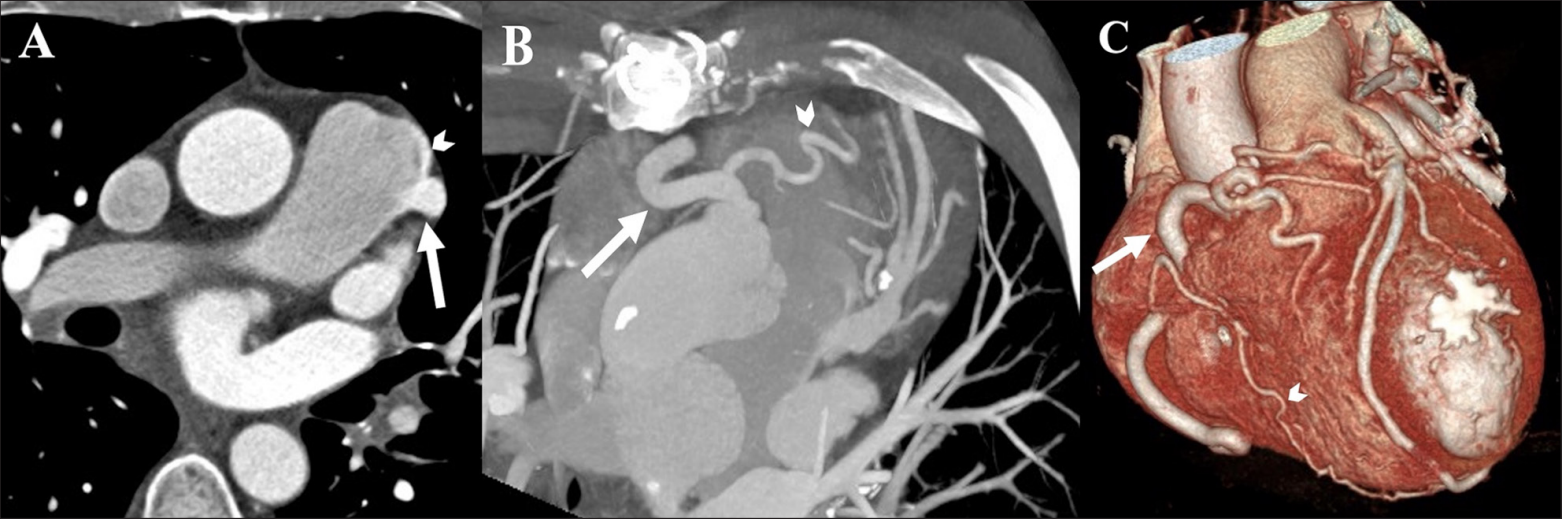


The cardiac CT showed an abnormal origin of a dilated left coronary artery (LCA) from the pulmonary artery (PA) (Figure 1A) and then followed its usual course towards the interventricular groove and branched into the left anterior descending artery (LAD) and left circumflex artery (LCX). A ‘jet’ appearance of contrast from the LCA into the pulmonary artery as a manifestation of retrograde flow was also seen (Figure 1A). Other findings included a dilated and tortuous right coronary artery (RCA) with collateral intercoronary arteries developed between the RCA and the LCA (Figure 1B, C). Furthermore, bronchial arteries arising from the descending aorta were also anastomosed to the anomalous LCA.

**Figure 2:**
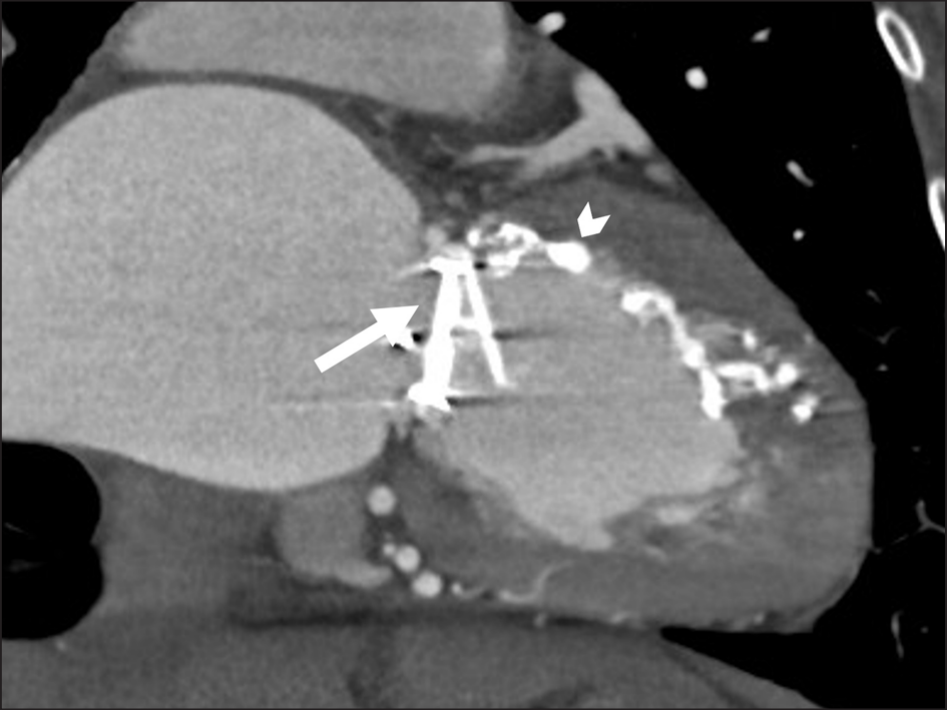


The CT demonstrated multiple areas of myocardial and subendocardial infarcts and a large left ventricle apex. A mitral mechanic valve was also depicted (Figure 2). These findings were consistent with an adult type of anomalous origin of the LCA from the pulmonary artery (ALCAPA) syndrome. The patient was treated only for her PAF by invasive intervention, and no surgical procedure was performed.

## Comments

ALCAPA syndrome is a rare congenital cardiac malformation that occurs in 1/300,000 births. It can be associated with other cardiac abnormalities such as atrial or ventricular septal defect and aortic coarctation. If not treated, 90% of children with this syndrome die in their first year of life from myocardial infarction.

At birth, the decreased pressure in pulmonary circulation leads to a steal phenomenon characterized by a reverse flow in the LCA that fails to supply the myocardium. Patients survive infancy by developing a large collateral circulation from the RCA and systemic arteries. The blood supply by collaterals is not always enough, especially in the subendocardial region, resulting in myocardial and subendocardial infarction resulting in malignant dysrhythmias and mitral dysfunction by prolapse and regurgitation.

ECG-gated CT angiography offers excellent spatial resolution to assess the collaterals and the coronary arteries. It can also be useful as a post-operative follow-up imaging. Fast cine magnetic resonance imaging demonstrates the steal phenomenon from the LCA into the PA. Surgical repair is the treatment of choice. However, cases of uncorrected ALCAPA syndrome have been described when the risk-benefit ratio of intervention is doubtful [1].
